# Patterns of antihypertensive and statin adherence prior to dementia: findings from the adult changes in thought study

**DOI:** 10.1186/s12877-019-1058-6

**Published:** 2019-02-14

**Authors:** Zachary A. Marcum, Rod L. Walker, Bobby L. Jones, Arvind Ramaprasan, Shelly L. Gray, Sascha Dublin, Paul K. Crane, Eric B. Larson

**Affiliations:** 1 Department of Pharmacy, School of Pharmacy, University of Washington, 1959 NE Pacific St, H375G, Box 357630, Seattle, WA 98195-7630 USA; 2 Kaiser Permanente Washington Health Research Institute, 1730 Minor Ave, Ste 1600, Seattle, WA 98101 USA; 3 Department of Psychiatry, University of Pittsburgh Medical Center, 3811 O’Hara St, Pittsburgh, PA 15213 USA; 4 Division of General Internal Medicine, School of Medicine, University of Washington, 325 Ninth Avenue, Box 359780, Seattle, WA 98104 USA

**Keywords:** Medication adherence, Dementia, Community dwelling, Older adults, Antihypertensives

## Abstract

**Background:**

Detecting patients with undiagnosed dementia is an important clinical challenge. Changes in medication adherence might represent an early sign of cognitive impairment. We sought to examine antihypertensive and statin adherence trajectories in community-dwelling older adults, comparing people who went on to develop dementia to those who did not.

**Methods:**

We analyzed data from Adult Changes in Thought (ACT), a population-based cohort study embedded within an integrated healthcare delivery system. Analyses included 4368 participants aged ≥65 years who had at least one follow-up visit. Research-quality dementia diagnoses were used to identify cases. We selected non-dementia control visits matched on age, sex, and study cohort that occurred at similar ACT follow-up time as the case’s dementia onset; we treated this as the index date. Participants were included if they were prevalent users of either a statin or antihypertensive medication on the first day of follow up – 3 years prior to the index date. Using prescription fill dates and days supply, we calculated daily binary medication availability measures for each participant (‘days covered’) over 3 years leading up to the index date. We used group-based trajectory models to identify patterns of antihypertensive and statin adherence, and used conditional logistic regression to examine associations between adherence trajectories and dementia.

**Results:**

Four trajectories were identified for antihypertensive users (292 cases, 3890 control visits), including near perfect (*n* = 1877, 36.6% cases, 45.5% controls), high (*n* = 1840, 43.2% cases, 44.1% controls), moderate (*n* = 365, 18.5% cases, 8.0% controls) and early poor adherence (*n* = 100, 1.7% cases, 2.4% controls). Odds of dementia was 3 times greater for those with moderate antihypertensive adherence compared to those with near perfect adherence (adjusted OR 3.0, 95% CI 2.0, 4.3). Four trajectories were identified for statin users (148 cases, 1131 control visits), including high (*n* = 1004, 75.0% cases, 79.0% controls), moderate (*n* = 192, 19.6% cases, 14.4% controls), early poor (*n* = 43, 2.0% cases, 3.5% controls), and delayed poor adherence (*n* = 40, 3.4% cases, 3.1% controls). No association was detected between statin adherence trajectories and dementia.

**Conclusions:**

Patterns of medication adherence may be useful to identify a subset of people at higher likelihood of developing dementia.

**Electronic supplementary material:**

The online version of this article (10.1186/s12877-019-1058-6) contains supplementary material, which is available to authorized users.

## Background

Only half of the more than 5 million people in the United States living with dementia have been diagnosed. [1] Detecting patients with undiagnosed cognitive impairment or dementia is important because earlier diagnosis may enable clinicians to improve management of comorbid medical conditions, among other benefits. Healthcare utilization patterns can offer important insights into patients’ cognitive status. For example, patients with undiagnosed dementia are more likely to “no-show” for scheduled appointments, to visit the emergency department, and to be hospitalized compared to those without dementia. [2] Changes in medication adherence may be an additional early indicator of cognitive impairment. [3] Studies have shown that cognitive abilities are important for optimal medication adherence in older adults. [4–9]

Decline in average global cognitive function may begin at least 10 years before clinical diagnosis of dementia, with the decline accelerated about 3 years before clinical diagnosis. [10] Given that cognitive decline characterizes dementia, one might expect that medication adherence might change well before onset of dementia. However, little is known about medication adherence patterns in the years immediately prior to dementia diagnosis. Identifying distinct adherence patterns in those who subsequently develop dementia could be useful in efforts to screen for early, or undiagnosed, cognitive impairment or dementia.

The proportion of days covered (PDC) method is a commonly used pharmacy claims-based measures of medication adherence since it was developed and endorsed by the Pharmacy Quality Alliance/National Quality Forum to be used in the Medicare Star Ratings. [11, 12] In the setting of possible cognitive impairment, using pharmacy claims to measure medication adherence offers an advantage over other measurement approaches such as self-report. Moreover, group-based trajectory modeling (GBTM) – a method derived from sociological research [13] – is increasingly being applied to medical research settings, including assessment of longitudinal medication adherence. [14, 15] While the average PDC is a static measure of adherence, GBTM is a dynamic measure and offers an alternative approach for measuring longitudinal medication adherence.

In this study, we used longitudinal data from a cohort of individuals without dementia at baseline in the Adult Changes in Thought (ACT) study. We used GBTM to examine patterns of adherence to antihypertensive medications and statins – two of the most commonly used medication classes in older adults – over 3 years in community-dwelling older adults, comparing people who went on to develop dementia versus those who did not.

## Methods

### Design, study setting, and participants

The research protocol for this population-based prospective cohort study was reviewed and approved by institutional review boards of Kaiser Permanente Washington (KPW, formerly Group Health Cooperative) and the University of Washington. Participants provided written informed consent. KPW is an integrated healthcare delivery system located in the Seattle, Washington area in the United States.

The Adult Changes in Thought (ACT) study enrolled 2581 community dwelling dementia-free participants randomly sampled from KPW members aged ≥65 in 1994–1996. [16] An additional 811 participants were enrolled from 2000 through 2003, and in 2005, the study began continuous enrollment to replace those who died or dropped out. Study details have been described in detail elsewhere. [17] Participants underwent assessment at study entry and every two years to evaluate cognitive function and collect demographic characteristics, medical history, health behaviors, and health status. ACT’s Completeness of Follow-Up Index (> 97%) has been exemplary. [18] In addition, information on participants’ health care utilization and medication dispensing were available from KPW electronic databases.

### Identification of dementia cases and non-dementia control visits

Participants were assessed with the Cognitive Abilities Screening Instrument (CASI) at study entry and subsequent biennial visits. CASI scores range from 0 to 100, with higher scores indicating better cognitive performance. Participants with scores ≤85 underwent a standardized diagnostic evaluation for dementia, including a physical and neurological examination, and neuropsychological tests. Results of these evaluations and laboratory and imaging records were then reviewed in a multidisciplinary consensus conference (procedures are described in detail elsewhere). [17] Diagnoses of dementia and probable or possible AD were made using research criteria. [19, 20] For each dementia case in our analysis, we matched non-dementia control visits – i.e., study visits occurring at a similar follow-up time as the case’s dementia onset (within +/− 1 year) from ACT subjects of the same age (within +/− 1 year), sex, and ACT study cohort but without dementia. Cases could have 1 to N matching controls, and controls could be matched to multiple cases. Cases without a matching control were excluded from analyses.

**Fig. 1:**
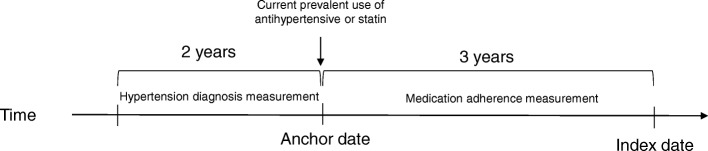
Study Design. ^a^ The index date was the date of dementia onset for cases, while for matched controls it was a study visit date at similar ACT follow-up time as the case’s dementia onset (within +/− 1 year). The anchor date was defined as three years before the index date. Participants in the antihypertensive analysis were also required to have at least one ICD-9 claim for a hypertension diagnosis in the 24 months prior to the anchor date. Computerized pharmacy dispensing data were used to identify people with prevalent use of antihypertensives or a statin as of the anchor date. Group-based trajectory modeling was employed to identify distinct patterns of medication adherence for antihypertensive and statin PDC data over the 3 years between anchor and index date

Our current analysis examined longitudinal trajectories of medication adherence for two types of chronic medications commonly used by older adults: antihypertensives and statins. We identified separate subgroups of ACT participants for each of these medication class analyses. All analyses focused on the 3-year period between an anchor date and an index date (Fig. 1). The index date was the date of dementia onset for cases and the time of the non-dementia control visit for matched controls. The anchor date was defined as three years before the index date.

Included ACT participants were required to have at least one follow-up study visit, to not report ever living in a nursing home, and to be continuously enrolled in KPW for at least 3.5 years prior to the index date. We required slightly more than 3 years prior enrollment to ensure capture of medication fills that closely precede (and overlap) the anchor date. In addition, since KPW’s pharmacy database did not capture days supply information until 1996, we limited analysis to index dates occurring after June 1999, allowing us to have complete days supply information for the analytic period of interest. Participants in the antihypertensive analysis were also required to have at least one ICD-9 claim for a hypertension diagnosis in the 24 months prior to the anchor date. Data through June 30, 2015 were included in these analyses.

### Medication adherence

Computerized pharmacy dispensing data were used to identify people with prevalent use of antihypertensives or a statin as of the anchor date. Using prescription fill dates and days supply, we calculated daily binary medication availability measures for each participant for 3 years leading up to the index date. We reduced the daily data by averaging consecutive 15-day windows over the 3-year period (using the logic of the proportion of days covered [PDC] adherence measure). Medication adherence was measured for the overall medication class (antihypertensive medication or a statin), rather than for individual medications or medication sub-classes. This decision reflects prescribing practices where these medications are used interchangeably within a given class and oftentimes more than one sub-class at the same time (i.e., for antihypertensives).

### Participant characteristics

Information about participant characteristics came from questionnaires administered at study visits or from KPW electronic health databases. Demographic factors included age at anchor date, sex, and years of education. Co-morbidity was measured using the Charlson risk score [21], including outpatient, inpatient, and emergency room utilization data for the 12 months prior to anchor date. Prior antihypertensive or statin use was expressed in years of use prior to anchor date.

### Statistical analysis

Descriptive statistics were calculated for demographic and health status variables as well as 3-year PDC values by antihypertensive medication or statin use. Further, we employed group-based trajectory modeling (GBTM) to identify distinct patterns of medication adherence for antihypertensive and statin PDC data over the 3 years between anchor and index date. [15] GBTM is increasingly being used to evaluate longitudinal medication adherence patterns. [13, 14] The overall goal of GBTM is to describe the course of an outcome for distinct sub-groups – in this case, medication adherence – over time; this is in contrast to static measures of medication adherence such as the average PDC that is often used. The average PDC is simply the mean number of days covered by a medication divided by the duration of the time period. GBTM produces two outputs not provided by using an average PDC: the longitudinal trajectories and the prevalence of trajectory membership. To compare the GBTM approach with the more traditional calculation of one-year PDC, we also calculated one-year incremental PDC values for antihypertensive and statin medications over the 3 years between anchor and index date. Analyses were conducted using *traj* in Stata 14 (StataCorp., 2015). [22]

First, models were fit separately for dementia cases and non-dementia controls and separately for each medication class. Each individual was assigned to one trajectory group based on the probabilities of individual membership in each trajectory group generated from the model. We used a censored normal model for the 15-day average PDC to accommodate the excess of zeroes and ones at the scale minimum and maximum. We then estimated models with 2 to 5 trajectories and selected the final number of trajectory groups based on the ability of the model to discriminate between trajectories, clinical interpretability, and having a reasonable sample size in each trajectory. [15] Trajectory groups are labeled based on appearance to aid interpretation.

To determine if dementia case status was more likely in certain trajectories, we fit a combined (cases and controls) model using GBTM. We used conditional logistic regression to examine associations between trajectories and dementia status, adjusting for comorbidity, education, and years of prior antihypertensive or statin use. Age, sex, and ACT study cohort were not adjusted for as they were matching variables. We intentionally did not adjust for changes in cognition given our hypothesis that such changes prior to dementia diagnosis could be impairing medication adherence. We are not aware of previous studies using GBTM to measure medication adherence in populations with neurodegenerative disorders. Regression models were estimated using SAS software, version 9.4 (SAS Institute, Inc., Cary, NC).

## Results

### Antihypertensive use

Characteristics of prevalent antihypertensive users were similar between dementia cases and all matched control visits (Table 1), with the exception of a smaller proportion of dementia cases who were female (69% vs. 80%). The 3-year mean PDC for antihypertensives was 0.88 (SD 0.15) for dementia cases (*n* = 292) and 0.92 (SD 0.13) for controls (*n* = 3890). The 1-year incremental mean PDC values for antihypertensives were largely unchanged over the 3 years between anchor and index date for dementia cases and controls (Table 2).

**Table 1:** Prevalent Antihypertensive and Statin User Characteristics

Antihypertensive Users	–	–
Variable, mean ± SD	Dementia, Cases^d^ (*n* = 292)	Non-Dementia, Controls (*n* = 3890)
Age (at anchor date^a^), years	82.3 ± 5.4	80.9 ± 4.9
Female sex, n (%)	201 (68.8)	3095 (79.6)
Years of education	14.0 ± 2.8	14.1 ± 2.9
Charlson risk score^b^	1.8 ± 2.1	1.6 ± 1.9
Years of antihypertensive use (prior to anchor date)	16.2 ± 9.2	16.3 ± 9.1
Proportion of Days Covered (PDC)^c^	0.88 ± 0.15	0.92 ± 0.13
Statin Users	–	–
Variable, mean ± SD	Dementia, Cases^e^ (n= 148)	Non-Dementia, Controls (n = 1131)
Age (at anchor date^a^), years	81.4 ± 5.2	79.8 ± 5.8
Female sex, n (%)	77 (52.0)	568 (50.2)
Years of education	14.3 ± 3.0	14.8 ± 3.0
Charlson risk score^b^	2.1 ± 1.9	2.0 ± 2.1
Years of statin use (prior to anchor date)	5.7 ± 4.1	5.4 ± 4.0
Proportion of Days Covered (PDC)^c^	0.83 ± 0.17	0.85 (0.17)

Abbreviations: SD, standard deviation

^a^The index date was the date of dementia onset for cases, while for matched controls it was a study visit date at similar ACT follow-up time as the case’s dementia onset (within +/− 1 year). The anchor date was defined as three years before the index date

^b^Based on outpatient, inpatient, and emergency room utilization data

^c^Defined over 3 years, from anchor date to index date

^d^Dementia sub-type prevalence for cases in antihypertensive model: 59% Alzheimer’s disease type, 10% vascular, 22% multiple etiologies, 9% other. Age-specific dementia and Alzheimer’s disease incidence rates from the ACT Study are similar to those found in many other studies^17^

^e^Dementia sub-type prevalence for cases in statin model: 50% Alzheimer’s disease type, 11% vascular, 28% multiple etiologies, 11% other. Age-specific dementia and Alzheimer’s disease incidence rates from the ACT Study are similar to those found in many other studies^17^

**Table 2:** One-year Incremental Proportion of Days Covered Values for Antihypertensive and Statin Adherence

		Antihypertensive Users		Statin Users	
Year of PDC Measurement^a^	Measures	Dementia, Cases (n = 292)	Non-Dementia, Controls (*n* = 3890)	Dementia, Cases (*n* = 148)	Non-Dementia, Controls (*n* = 1131)
Year − 3 to − 2	Mean ± SD	0.90 ± 0.16	0.93 ± 0.12	0.84 ± 0.18	0.87 ± 0.15
	Min, Max	0.08, 1.0	0.01, 1.0	0.10, 1.0	0.05, 1.0
	Median	0.96	0.98	0.91	0.92
Year − 2 to − 1	Mean ± SD	0.88 ± 0.20	0.92 ± 0.16	0.83 ± 0.20	0.84 ± 0.22
	Min, Max	0,1.0	0, 1.0	0, 1.0	0, 1.0
	Median	0.96	0.98	0.89	0.92
Year − 1 to 0	Mean ± SD	0.88 ± 0.20	0.91 ± 0.19	0.81 ± 0.23	0.83 ± 0.24
	Min, Max	0, 1.0	0, 1.0	0, 1.0	0, 1.0
	Median	0.96	0.98	0.89	0.91

Abbreviations: PDC, proportion of days covered

^a^Time period between anchor date and index date

Among antihypertensives, sub-class use over the 3-year follow-up period was similar between dementia cases and controls with a few exceptions (Additional file 1: Table S1). Compared to controls, a smaller proportion of dementia cases used angiotensin receptor blockers (“ARBs”; 10% vs. 16%) and calcium channel blockers (42% vs. 47%). Conversely, a greater proportion of dementia cases used beta-blockers (67% vs. 63%).

**Fig. 2:**
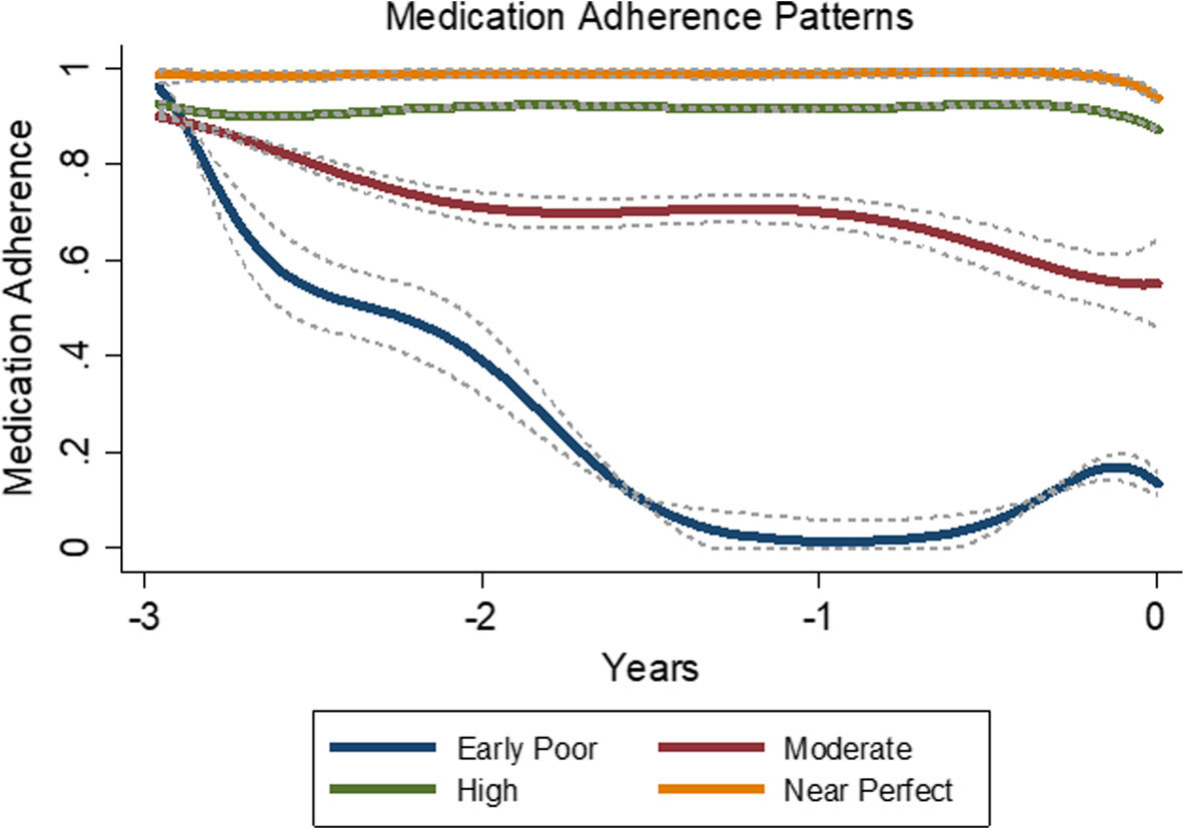
Antihypertensive Adherence Trajectories over 3 Years: Dementia Cases and Non-Dementia Controls Combined (*N* = 4182). ^a^ Each individual was assigned to one trajectory group based on the probabilities of individual membership in each trajectory group generated from the model. We used a censored normal model for the 15-day average PDC to accommodate the excess of zeroes and ones at the scale minimum and maximum. Group-based trajectory modeling suggested a 4-group solution for antihypertensive users. The 4 medication adherence trajectories and their 95% confidence intervals are displayed in the Figure. Trajectory groups are labeled based on appearance to aid interpretation. ^b^ Trajectory group prevalence included near perfect (*n* = 1877, 37% cases, 46% controls), high (*n* = 1840, 43% cases, 44% controls), moderate (*n* = 365, 18% cases, 8% controls) and early poor adherence (*n* = 100, 1.7% cases, 2.4% controls)

GBTM suggested a 4-group solution for separate models of both dementia cases and non-dementia controls using antihypertensives. Using the combined model (cases and controls), four medication adherence trajectories were identified for antihypertensive users (292 cases, 3890 control visits, *N* = 4182 total), including near perfect (*n* = 1877, 37% cases, 46% controls), high (*n* = 1840, 43% cases, 44% controls), moderate (*n* = 365, 18% cases, 8% controls) and early poor adherence (*n* = 100, 1.7% cases, 2.4% controls) (Fig. 2). An association was detected between adherence patterns and dementia (omnibus *p*-value < 0.001), with odds of dementia estimated to be significantly higher only among those with moderate antihypertensive adherence (relative to the other groups). For example, odds of dementia was 3 times greater for those with moderate antihypertensive adherence compared to those with near perfect adherence (adjusted OR 3.0, 95% CI 2.0, 4.3), after adjusting for comorbidity, education, and years of prior antihypertensive use (Table 3). Odds of dementia was similar among those with near perfect, high, and early poor adherence.

**Table 3:** Conditional Logistic Regression Models for the Association between Antihypertensive and Statin Adherence Patterns and Dementia

	Adjusted Odds Ratio, 95% Confidence Interval^a^
Antihypertensive Adherence Patterns	–
Near perfect adherence	1.0 (*Reference*)
High adherence	1.2 (0.9–1.6)
Moderate adherence	3.0 (2.0–4.3)
Early poor adherence	1.0 (0.4–2.6)
Statin Adherence Patterns	–
High adherence	1.0 (*Reference*)
Moderate adherence	1.5 (0.9–2.4)
Early poor adherence	0.6 (0.2–1.9)
Delayed poor adherence	1.4 (0.5–3.7)

^a^Adjusted for comorbidity, education, and years of prior antihypertensive or statin use. Age, sex, and ACT study cohort were matching variables

### Statin Use

Characteristics of prevalent statin users were similar between dementia cases and matched control visits (Table 1). Antihypertensive and statin users displayed similar characteristics, except that a higher proportion of statin users were male. The 3-year mean PDC for statins was 0.83 (SD 0.17) for dementia cases (*n* = 148) and 0.85 (SD 0.17) for controls (*n* = 1131). The 1-year incremental mean PDC values for statins were largely unchanged over the 3 years between anchor and index date for dementia cases and controls (Table 2).

**Fig. 3:**
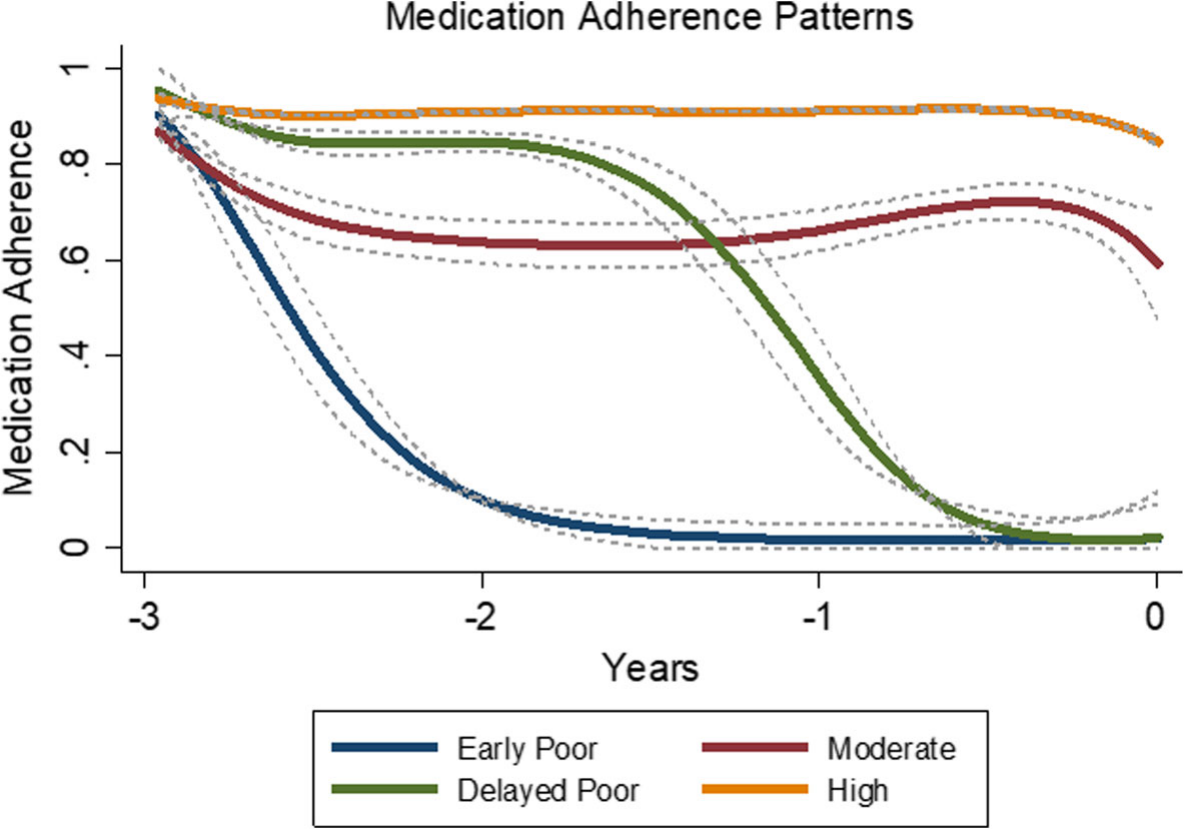
Statin Adherence Trajectories over 3 Years: Dementia Cases and Non-Dementia Controls Combined (*N* = 1279). ^a^ Each individual was assigned to one trajectory group based on the probabilities of individual membership in each trajectory group generated from the model. We used a censored normal model for the 15-day average PDC to accommodate the excess of zeroes and ones at the scale minimum and maximum. Group-based trajectory modeling suggested a 4-group solution for statin users. The 4 medication adherence trajectories and their 95% confidence intervals are displayed in the Figure. Trajectory groups are labeled based on appearance to aid interpretation. ^b^ Trajectory group prevalence included high (*n* = 1004, 75% cases, 79% controls), moderate (*n* = 192, 20% cases, 14% controls), early poor (*n* = 43, 2.0% cases, 3.5% controls), and delayed poor adherence (*n* = 40, 3.4% cases, 3.1% controls)

GBTM suggested a 4-group solution for separate models of both dementia cases and non-dementia controls using statins. Using the combined model (cases and controls), four medication adherence trajectories were identified for statin users (148 cases, 1131 controls, *N* = 1279 total), including high (*n* = 1004, 75% cases, 79% controls), moderate (*n* = 192, 20% cases, 14% controls), early poor (*n* = 43, 2.0% cases, 3.5% controls), and delayed poor adherence (*n* = 40, 3.4% cases, 3.1% controls) (Fig. 3). No significant association (omnibus *p*-value = 0.256) was detected between statin adherence patterns and dementia (Table 3).

## Discussion

In this prospective community-based cohort study, we tested whether there were differences in antihypertensive and statin medication adherence patterns between people who went on to develop dementia versus those who did not. We found some evidence of differences among antihypertensive users such that the odds of dementia was greatest for those with moderate antihypertensive adherence over the 3-year period. We did not find an association between statin medication adherence trajectory and subsequent dementia status, though our power was lower to detect such an association since antihypertensive use was more common than statin use in our cohort. One potential reason for our findings is that statins could be considered easier to take. However, in a previous report we found that the prevalence of medication non-adherence was similar for antihypertensives and statins in a nationally representative sample of older adults. Thus, we doubt this factor explains our findings. [23]

The proportion of days covered (PDC) method is a commonly used pharmacy claims-based measures of medication adherence since it is used in the Medicare Star Ratings. [11, 12] However, GBTM is increasingly being applied to medical research settings, including assessment of longitudinal medication adherence. [14, 15] As a dynamic measure, GBTM offers an alternative approach for measuring longitudinal medication adherence and is more aligned with measuring a complex health behavior such as adherence. As a result, GBTM can provide insights into key behavioral sub-groups for identification and intervention. For example, the 3-year mean PDC for antihypertensive use in our study was 0.88 and 0.92 for dementia cases and controls, respectively. In addition, the 1-year mean PDC values for antihypertensive use over the 3-year observation period were largely unchanged over time and showed very little differences between dementia cases and controls. These average results alone would lead one to conclude that antihypertensive adherence was high in this sample without any insights into possible sub-groups with differentially poor adherence. Conversely, applying GBTM to antihypertensive users identified a potentially important sub-group – moderate adherence – that accounted for 9% antihypertensive users. This pattern started with high adherence and slowly declined over the 3 years leading up to the index date, ultimately ending up with an adherence less than 60%. This information – the shape of the pattern and the relative prevalence of group membership – is not provided from the PDC.

Not surprisingly, medication adherence changes may be an early indicator of cognitive impairment. Mizokami et al. evaluated in a cross-sectional study the relationship between medication adherence and cognitive decline using instrumental activities of daily living (IADL) and cognitive function (via the Mini-Mental State Examination) in a hospital setting in Japan. [3] They found that lower IADL scores in *‘responsibility for own medications’* might be an effective index for predicting early-stage cognitive dysfunction in older adults. However, this study was limited by its cross-sectional design. Although we did not assess IADLs in our study, medication non-adherence (as measured by pharmacy claims) could serve as a proxy for having difficulty with medication management and thus indicate a need for more careful monitoring of cognitive function. Furthermore, polypharmacy is an important factor in medication adherence for older adults. [24] Comorbidity as measured by the Charlson score may be considered a proxy of medication regimen complexity. As shown in Table 1, these scores did not differ between dementia cases and controls. We thus doubt that differences in regimen complexity explain our findings.

While we used pharmacy claims as the measure of medication adherence for this study, claims data may be unavailable to clinicians. [25] Franklin et al. showed that electronic health record (EHR) data provide good predictions of medication adherence trajectories (i.e., statins, antihypertensives, and oral antidiabetic drugs) in a Medicare Advantage sample. [25] Ideally, multiple sources of medication adherence data would be integrated into the patient’s health record for enhanced clinical decision-making and improved patient outcomes. [26] Regardless of the data source, longitudinal trajectories provide a clinically intuitive summary of a patient’s medication use patterns and can be created using already-collected data. Future research is needed to evaluate implementation of medication adherence data into clinical practice for a broad range of applications, especially for improving the detection of early, or undiagnosed, cognitive impairment or dementia.

Strengths of this study include the prospective community-based design, the large sample with minimal attrition, extensive longitudinal medication records based on computerized pharmacy data for prescription fills, and prospective ascertainment of research-quality cases. Several limitations should be acknowledged. First, while GTBM provides visually intuitive output, it is important to note its limitations as a statistical method in which individuals are assigned to one trajectory group based on the probabilities of individual membership in each trajectory group generated from the model. In other words, as with all statistical models, the trajectories are statistical approximations and not actual entities. As such, we used GBTM in this study to identify potential fruitful areas for future research. Moreover, the objective of this study was not to assess causality between medication adherence patterns and dementia. Thus, it is unclear if suboptimal antihypertensive adherence is associated with dementia, or if suboptimal antihypertensive adherence is a sign of early cognitive decline. Future research is needed to disentangle the temporal association between cognitive decline, medication adherence, and dementia. Such research should be mindful of modeling cognitive decline in non-linear manner. The ACT Study also does not categorize people as having mild cognitive impairment (MCI) or not. Other studies may be able to address how medication adherence might differ from normal cognition to MCI to dementia. Second, we assessed medication adherence on the overall therapeutic class level and not for individual drugs. For the antihypertensive analysis, this approach may have led to an overestimate of adherence. Third, while we excluded people who lived in nursing homes and thus had access to assistance with medication use, we were unable to measure receipt of caregiving, which could have an impact on medication adherence. Future research should examine the association between caregiver assistance with medication management and health outcomes in older adults. Moreover, pharmacy claims serve as a proxy for medication adherence. As such, it was possible to measure only medication supply and not actual medication taking, although this approach is commonly used in quality assessment for medication adherence. Fourth, we were limited in our sample size of dementia cases who were prevalent statin users and thus may have had limited power to detect associations between odds of dementia and statin adherence trajectories. This could potentially explain our finding of a statistically significant association between an antihypertensive medication adherence trajectory and odds of dementia but not a statin adherence trajectory. Fifth, some participants may have had CASI scores above our threshold despite mild dementia. In that scenario, we would have underestimated the number of dementia cases and overestimated the number of non-dementia cases, leading to potential bias due to misclassification. Finally, our results may not be generalizable to other healthcare settings as our analysis was conducted in integrated healthcare delivery system where efforts are made to improve medication adherence (e.g., automatic refills, 90-day supply).

## Conclusions

In conclusion, we found that the odds of dementia was greater for people with moderate antihypertensive adherence over 3 years compared to those with other adherence patterns. An association between statin medication adherence trajectory and subsequent dementia status was not detected. Medication adherence has potential to be useful in detecting dementia, and future studies are needed to evaluate the potential role of medication non-adherence as a tool to detect early, or undiagnosed, cognitive impairment and dementia.

## Additional file

Additional file 1: **Table S1.** Prevalence of Antihypertensive Use by Sub-Class across 3 Years. (DOCX 14 kb)

## Abbreviations

ACT: Adult Changes in Thought
CASI: Cognitive Abilities Screening Instrument
EHR: electronic health record
GBTM: group-based trajectory modeling
IADL: instrumental activities of daily living
ICD: international classification of diseases
KPW: Kaiser Permanente Washington
PDC: proportion of days covered
