# Supplementary material for: Patterns of antihypertensive and statin adherence prior to dementia: findings from the adult changes in thought study
Source: BMC Geriatr. 2019 Feb 14;19:41. doi: 10.1186/s12877-019-1058-6 (PMC6376744; doi:10.1186/s12877-019-1058-6)
Supplement: Supplementary file 1 — Table S1. Prevalence of Antihypertensive Use by Sub-Class across 3 Years. (DOCX 14 kb) [file 12877_2019_1058_MOESM1_ESM.docx]

**Table S1.** Prevalence of Antihypertensive Use by Sub-Class across 3 Years

| **Antihypertensive Sub-Class, n (%)** | **Dementia,**  **Cases (n=292)** | **Non-Dementia, Controls (n=3890)** |
| --- | --- | --- |
| Beta blockers | 196 (67.1) | 2461 (63.3) |
| Thiazide diuretics | 174 (59.6) | 2356 (60.6) |
| Angiotensin converting enzyme inhibitors | 162 (55.5) | 2179 (56.0) |
| Calcium channel blockers | 122 (41.8) | 1826 (46.9) |
| Non-loop diuretics | 41 (14.0) | 546 (14.0) |
| Angiotensin II receptor blockers | 28 (9.6) | 638 (16.4) |
| Adrenergic (central) agents | 8 (2.7) | 121 (3.1) |
| Peripheral vasodilators | 7 (2.4) | 78 (2.0) |
| Renin inhibitors | 0 (0) | 1 (0) |
